# The Coexistence of Psychiatric Disorders and Intellectual Disability in Children Aged 3–18 Years in the Barwani District, India

**DOI:** 10.1155/2013/875873

**Published:** 2013-05-07

**Authors:** Ram Lakhan

**Affiliations:** School of Health Sciences, Jackson State University, 350 West Woodrow Wilson Drive, Jackson Medical Mall, Suite NO. 320, Jackson, MS 39213, USA

## Abstract

*Background*. The coexistence of psychiatric disorders in people with intellectual disability (ID) is common. This study determined the prevalence of psychiatric disorders in children with ID in Barwani, India. *Method*. A total of 262 children with ID were evaluated for psychiatric disorders using the diagnostic criteria outlined in the International Classification of Diseases (ICD-10). *Results*. Psychiatric disorders appeared in study participants at the following rates: attention deficit hyperactivity disorder (ADHD), 6.5%; autism, 4.2%; anxiety, 2.7%; bipolar disorder, 1.1%; delusional disorder, 0.8%; depression, 2.3%; obsessive-compulsive disorder, 0.8%; schizophrenia, 1.9%; enuresis, 10.3%; epilepsy, 23.7%; and behavioral problems, 80.9%. The prevalence of psychiatric disorders was statistically higher in severely intellectually disabled children (IQ ≤ 49) than mildly intellectually disabled children (IQ ≥ 50). *Conclusions*. There is a higher prevalence of psychiatric disorders in children with ID when their IQ ≤ 49 compared with ID children whose IQ ≥ 50.

## 1. Introduction 

Intellectual disability (ID), previously referred to as mental retardation, is most often associated with other medical and psychiatric conditions such as cerebral palsy, epilepsy, Down syndrome, fragile X syndrome, attention deficit hyperactivity disorder (ADHD), autism, and other emotional and behavioral disorders. The coexistence of psychiatric disorders occurring in people with ID is not uncommon. The study of intellectual disability falls within the field of psychiatry, in which dual diagnoses have historically been common. However, specifically investigating the coexistence of psychiatry disorders among people with ID has only recently begun [1–3]. Compared with the general population, people with ID have a higher prevalence of psychiatry disorders [4, 5], ranging from 10% to 80% [6, 7]. Other epidemiological studies have reported similar prevalence rates [8–13]. 

For the past few decades, the psychiatric community in developed countries, like England and Australia, has done more to identify the psychiatric needs of people with ID, which has allowed doctors to provide these patients with appropriate mental health services [7, 14–16]. In India dual diagnosis of psychiatric disorders in people with ID has gradually become more common since implementation of the Persons with Disabilities Act in 1995. However, making further efforts to identify people in India with ID who also suffer from psychiatric disorders is necessary to plan for and provide comprehensive intervention for their well-being [17]. Identifying psychiatric disorders in people with ID is a highly specialized area for professionals because the symptoms of psychiatric disorders in people with ID do not always resemble the symptoms of the same psychiatric disorders in the general population. Furthermore, poor cognitive abilities may overshadow other psychiatric disorders in children with ID, preventing accurate diagnoses of coexisting conditions [18, 19].

*Objective*. To determine the prevalence of coexisting psychiatry disorders in children with ID ranging in age from 3 to 18 years in Barwani.

*Aim.* To estimate the prevalence of coexisting psychiatric disorders among children with ID identified in a cross-sectional study as part of a community-based rehabilitation project in the Barwani block of Barwani District in Madhya Pradesh. 

## 2. Method 

### 2.1. Demographics and Sampling

Ashagram Trust (AGT) is a nongovernment organization located in Barwani that provides medical and rehabilitation services to the people of Barwani and its surrounding districts. Barwani is one of the poorest districts in India [20]. It has two populations: tribal (68%) and nontribal (32%). Of the entire district, 53% of the population lives below the poverty line. In general, the tribal population is more disadvantaged in the district than the nontribal population [21]. With financial help from Action Aid, AGT implemented a community-based rehabilitation (CBR) project, spanning from 1999 to 2010, in 63 villages of the Barwani block. In 2001, a total of 10,909 households, comprising 63,789 people (2001 census), in 51 villages were surveyed door-to-door to identify children with ID. All identified children were recruited so that they could receive proper medical, rehabilitation, educational, and vocational support. Written consent was obtained at two levels. First, consent to conduct the screening survey was obtained from each village's politically elected leader (Sarpanch). Consent was also obtained from the heads of each family (parents or grandparents) to assess the child for ID and coexisting disorders and receive comprehensive intervention under the CBR project. An ethical committee comprising members of the funding organization *Action Aid*, AGT, and the community approved the study. From this survey, 262 children ranging in age from 3 to 18 years were identified as having ID. Children with ID who developed psychiatric disorders during the course of the study were also included in the study.

### 2.2. Diagnosis of Intellectual Disability

Children identified in the cross-sectional study were further evaluated by two professionals specializing in the assessment of mental retardation (MR). These professionals were employed by the CBR project at the time (one is the author of this paper). Diagnoses of ID were made according to IQ classification, following the criteria outlined in the ICD-10. The ICD-10 criteria are popular [22] and widely used across member countries of the World Health Organization to diagnose people with mental and behavioral disorders [23]. Two tests, a developmental screening test (DST) developed by the National Institute for the Mentally Handicapped (NIMH) in India and an Indian adaptation of the Vineland Social Maturity Scale (VSMS), were used in the first round of assessment. The outcomes of these tests were used to calculate the IQ. The DST provided the developmental quotient (DQ) value, while the VSMS provided the social quotient (SQ) value. The average of these two values (DQ and SQ) is considered a person's IQ. Most of the study participants were also given an adaptation of the Stanford-Binet intelligence test, called the Binet-Kamat Test, which better reflects conditions in India [24]. A number of previous studies have supported the value of these tests, concluding that their psychometric properties are appropriate for making diagnoses [25, 26]. Furthermore, the NIMH in India recommends these tests for diagnosing people with ID. 

### 2.3. Diagnosis of Psychiatric Disorders

All cases evaluated by MR professionals were referred to a psychiatrist for psychiatric evaluation and a physician for physical examination. When cerebral palsy was found, the child was sent to an orthopedic surgeon. Laboratory testing was also done to determine chromosomal abnormalities. In each case, psychiatrists used clinical observations, mental state examinations, parental interviews, and reviews of previous consultations to make a psychiatric diagnosis, incorporating the ICD-10 criteria. Because epilepsy shares characteristics of both neurological and psychiatric disorders [27], we categorized it with other psychiatric disorders in this study. Children with ID who developed psychiatric disorders during the time frame of the project were also included in the study.

Occasionally, communication barriers arose because of the tribal dialect. Most professionals on the CBR team were able to understand tribal dialect, but they were unable to respond in a dialect that could be understood. Communication was also difficult with some nontribal parents. In situations where communication was a problem, community-based rehabilitation workers (CBRWs) interpreted between psychiatrists, MR professionals, and parents. Most cases were brought to the AGT psychiatric clinic, but a few could not reach the clinic because of inadequate transportation. In these situations, children were evaluated in rural camps within their own or nearby villages.

*Statistical Analysis*. SPSS Statistics software, student version 21, was used for data analysis. The prevalence of psychiatry disorders in the study's 262 children with ID was reported using frequencies and percentages. Chi-square tests were performed to compare the prevalence of psychiatric disorders with independent variables, such as the severity of ID (severe intellectual disability (SID; IQ ≤ 49) and mild intellectual disability (MID; IQ ≥ 50)), gender, poverty level, and population group (tribal and non-tribal). Because the “psychiatric group” disorders had expected count values less than five, the chi-square test was performed with the Yates correction. 

## 3. Result

Of the total ID population (*n* = 262), 17 (6.5%) were found to have ADHD, 11 (4.2%) were autistic, 7 (2.7%) showed anxiety, 3 (1.1%) had bipolar affective disorder, 2 (0.8%) had delusional disorder, 6 (2.3%) had depression, 2 (0.8%) had obsessive-compulsive disorder, 5 (1.9%) had schizophrenia, 27 (10.3%) had enuresis, 62 (23.7%) had epilepsy, and 212 (80.9%) showed behavioral problems. Psychiatric disorders coexisted within all five categories of ID (Table 1). However, children with profound ID appeared to only have epilepsy and behavioral problems. 

The number of disorders per child in each ID category was also examined. In the Borderline ID group, we found 1/5 disorders per child. However, the Mild and Moderate ID categories both had a ratio of 1.27 disorders per child, while the Severe ID group was calculated to have 1.65 disorders per child. The Profound ID group was calculated to have 1.47 disorders per child. Overall, behavioral problems were considerably more prevalent than other psychiatric disorders in all ID categories except Borderline ID. 

For comparison purposes, the rarely occurring psychiatric disorders of ADHD, autism, anxiety, bipolar disorder, delusional disorder, depression, obsessive-compulsive disorder, and schizophrenia were grouped together for analysis (psychiatric group). All cases of ID were grouped into categories of SID and MID. Upon comparing the SID and MID groups, we found that the prevalences of psychiatric group disorders *χ* ^2^ = 0.3*, *P* = .03) and epilepsy (*χ* ^2^ = 27.6, *P* < .001) were significantly higher in children with SID, while the prevalences of enuresis (*χ* ^2^ = 0.5, *P* = .31) and behavioral problems (*χ* ^2^ = 1.3, *P* = .25) were not statistically different between the two ID groups. The prevalences of psychiatric group disorders (*χ* ^2^ = 0.2*,  *P* = .54), enuresis (*χ* ^2^ = 1.7, *P* = .13), and behavioral problems (*χ* ^2^ = 0.1, *P* = .47) were not statistically different between male and female participants, but epilepsy was found to be more prevalent among male participants than females (*χ* ^2^ = 7.4, *P* = .005). Enuresis was found to be statistically more prevalent among poor children (*χ* ^2^ = 4.1, *P* = .03), but no differences in prevalence were found for psychiatric group disorders (*χ* ^2^ = 0.1*,  *P* = .91), behavioral problems (*χ* ^2^ = 0.0, *P* = .57), and epilepsy (*χ* ^2^ = 0.8, *P* = .45) between poor children and children who were not poor. The prevalences of enuresis (*χ* ^2^ = 7.2, *P* = .006) and behavioral problems (*χ* ^2^ = 4.1, *P* = .03) were higher among the tribal population, but psychiatric group disorders (*χ* ^2^ = 0.2*,  *P* = .50) and epilepsy (*χ* ^2^ = 0.1, *P* = .43) showed no significant difference in prevalence between tribal and nontribal populations (Table 2).

## 4. Discussion 

In this study, we found a higher prevalence of psychiatric disorders in children with ID having an IQ ≤ 49, an outcome that is consistent with the findings presented in Gillberg et al. (1986) [28]. Among children with an IQ ≤ 49, those with IQ between 49 and 35 had the highest rates of psychiatric disorders. Other than epilepsy and behavioral problems, none of the disorders from “psychiatric group” were found in children whose IQ ≤ 20. One possible explanation for this result is that diagnostic difficulty increases with the severity of a person's ID [6, 12, 13, 29]. 

The findings of our study related to psychiatric disorders in the tribal population are consistent with a prior study conducted on an Indian population by Kishore et al. (2004) [17]. That study found that children living in poor families and children belonging to the tribal community have a higher prevalence of enuresis, which may arise from improper care, improper training about personal care, activities of daily living, social exposure, and a lack of stimulation [30]. Our study also showed that behavioral problems are higher in the tribal population group, indicating that behavioral problems and enuresis could have similar contributing factors within this population. 

The prevalence of enuresis between males and females in this study was not found to be different. However, a previous study from 1986 indicated that males are likely to have a higher prevalence of enuresis than females, with 16% of males having the condition in that study, compared with 12% of females (*P* < .01) [31]. Enuresis is approximately twice as common in boys as in girls in the normal population [32]. Family history of enuresis, lower socioeconomic status, poor self-image, and diminished academic achievements are considered to be risk factors for enuresis [33]. In our study, factors like less gender discrimination, poverty, and simple lifestyles may have contributed to the equal prevalence of enuresis between boys and girls in the study population. 

The prevalence of epilepsy was not found to be affected by poverty level (poor versus not poor) and population group (tribal versus non-tribal), but it was found to be significantly higher in males than females. This finding is consistent with the normal population, in which studies have reported a marginally lower prevalence of epilepsy in females [34]. In our study, a significantly higher rate of epilepsy was found in children with severe and profound ID (IQ ≤ 49), which has previously been reported as the most common disorder in people with severe ID [35].

People with ID are vulnerable because of their limited cognitive abilities. Most of them need assistance to obtain and benefit from health services. When people with ID have additional psychiatric disorders, they become even more dependent on others and may urgently require mental health services [36]. Treating coexisting psychiatric disorders may favorably affect rehabilitation intervention in people with ID. Therefore, it is important to assess people with ID to identify any coexisting psychiatric disorders. A study by Mohr et al. (2002) [37] highlighted the benefits of diagnosing these coexisting conditions: they reported successful social rehabilitation of people with ID that had been concurrently diagnosed and treated for psychiatric disorders.

## 5. Study Limitation 

This study has several limitations. The sample size was very small. In addition standardized tests were used for ID diagnoses, but in some cases standardized tests were not used to diagnose psychiatric disorders, resulting in diagnoses made solely on the basis of clinical judgments by psychiatrists. 

Senior-level psychiatrists and lower-level trainee psychiatrists both provided their services in evaluating ID cases. However, the individual clinical abilities and biases of each psychiatrist might have impacted the results. Furthermore, the language barrier between clients and professionals was also a potential factor, which might have affected the clinical decisions made by professionals. Another limitation of the study was that the psychiatric unit could not maintain proper records in all cases; some of the case files did not contain complete information about the subjects or their clinical examinations. Thus, there exists the potential that some people who had psychiatric disorders were excluded in the study analysis. 

## 6. Conclusion 

This study shows that psychiatric disorders are not uncommon in people with intellectual disability. Thus, it is very important to properly diagnose any coexisting psychiatric disorders so that the appropriate services can be provided.

## Figures and Tables

**Table 1:** Prevalence of psychiatric disorders broken down by different categories of ID.

	Borderline	Mild	Moderate	Severe	Profound	
Psychiatric disorders/IQ level	5 (1.9%)	79 (30.2%)	100 (38.2%)	63 (24.0%)	15 (5.7%)	Total 262 (100%)
	(IQ > 70)	(IQ 69–50)	(IQ 49–35)	(IQ 34–20)	(IQ < 20)	
ADHD	0	6	9	2	0	17 (6.5%)
Anxiety	0	5	1	1	0	7 (2.7%)
Autism	0	3	6	2	0	11 (4.2%)
Bipolar affective disorder	0	0	1	2	0	3 (1.1%)
Delusional disorder	0	1	1	0	0	2 (0.8%)
Depression	1	3	2	0	0	6 (2.3%)
Obsessive compulsive disorder	0	2	0	0	0	2 (0.8%)
Schizophrenia	0	0	4	1	0	5 (1.9%)
Enuresis	0	7	11	9	0	27 (10.3%)
Epilepsy	0	3	18	32	9	62 (23.7%)
Behavior problems	0	70	74	55	13	212 (80.9%)

**Table 2:** The association of psychiatric disorders with different variables: severity of ID, gender, poverty level, and population group. Values are given for *χ* ^2^ statistics and their corresponding *P* values.

Variable		*N*		*N*	*χ* ^2^	*P* value
Severity of ID: SID versus MID						
Psychiatric group**	SID	32	MID	21	0.3*	.03
Enuresis	SID	20	MID	77	0.5	.31
Beh. problems	SID	142	MID	72	1.3	.25
Epilepsy	SID	59	MID	3	27.6	<.001
Gender: female versus male						
Psychiatric group**	F	24	M	28	0.2*	.54
Enuresis	F	16	M	11	1.7	.13
Beh. problems	F	102	M	112	0.1	.47
Epilepsy	F	20	M	42	7.4	.005
Poverty level: poor versus not poor (NP)						
Psychiatric group**	P	43	NP	10	0.1*	.91
Enuresis	P	25	NP	2	4.1	.03
Beh. problems	P	165	NP	49	0.0	.57
Epilepsy	P	47	NP	15	0.8	.45
Population group: tribal versus nontribal (NT)						
Psychiatric group**	T	32	NT	21	0.2*	.50
Enuresis	T	21	NT	6	7.2	.006
Beh. problems	T	108	NT	106	4.1	.03
Epilepsy	T	32	NT	30	0.1	.43

*Chi-square statistic with the Yates correction.

**Psychiatric group comprises ADHD, autism, anxiety, bipolar affective disorder, delusional disorder, depression, obsessive-compulsive disorder, and schizophrenia.
